# Carbon Footprint of Childhood Diets—A Secondary Analysis of Population-Based Studies

**DOI:** 10.1016/j.cdnut.2026.107705

**Published:** 2026-04-21

**Authors:** Tinna Odinsdottir, Ragnhildur Gudmannsdottir, Rachel Novotny, Birna Thorisdottir, Inga Thorsdottir

**Affiliations:** 1 Faculty of Food Science and Nutrition, School of Health Sciences, University of Iceland and Unit for Nutrition Research, Health Science Institute, University of Iceland, Reykjavík, Iceland; 2 Department of Human Nutrition, Food and Animal Sciences, College of Tropical Agriculture and Human Resilience, University of Hawai’i at Mānoa, Honolulu, HI, United States; 3 Department of Nutrition, Exercise and Sports (NEXS), Section for Nutrition and Health, University of Copenhagen, Copenhagen, Denmark

**Keywords:** children, dietary intake, carbon footprint, age trends, food-based dietary guidelines, sustainable diet, life cycle assessment

## Abstract

**Background:**

Food-based dietary guidelines (FBDG) aim to support health and reduce the environmental impacts, including the dietary carbon footprint (CF). Children’s dietary CF is largely unknown.

**Objectives:**

The aim of this study was to estimate the CF of children’s diets, identify the main food groups contributing to CF across different age groups, and to assess how adherence to FBDG might influence CF.

**Methods:**

The study included participants in 5 age groups (3, 5, 6, 9, and 15 y; *N* = 943) randomly selected from the capital area or nationwide during 2003–2012 in Iceland. Dietary intake was assessed using 3-d food records for 3-, 5-, and 6-y-olds and two 24-h recalls for 9- and 15-y-olds. Dietary CF was estimated using life cycle assessment data from the Danish CONCITO Big Climate Database. To assess how adherence to the Nordic FBDG alters dietary CF, comparison with the CF of recommended amounts of food groups, adjusted to children, was done.

**Results:**

Mean total dietary CF (kg CO_2_-eq/d) was 2.9–3.2 for 3-, 5-, and 6-y-olds and 4.1 for 9-y-olds; among 15-y-olds, it was 4.4 and 5.4 for girls and boys, respectively. Meat was the main contributor to CF across all age groups, followed by dairy products. Discretionary foods (e.g., sweets, snacks, and soft drinks) ranked third among 6-, 9-, and 15-y-olds. Fruits and vegetables contributed little to CF, as did fish, except in the 2 youngest age groups. Comparison with FBDG estimated for children showed that a closer adherence to FBDG would decrease CF.

**Conclusions:**

Meat and dairy were the largest contributors to dietary CF, with discretionary foods ranking third among children aged ≥6 y upward. The results highlight the potential positive climate impact of transitioning toward more plant-based diets and adhering to the FBDG.

## Introduction

Food preferences and dietary behaviors are developed early in life [[1], [2], [3], [4], [5]]. Early exposure to healthy and sustainable diets can, therefore, promote lifelong wholesome eating patterns with a low environmental impact. This highlights the importance of understanding the environmental impact of children’s diets and determining where improvements can be made.

Food-based dietary guidelines (FBDG) have traditionally focused on ensuring adequate nutrient intake to support health. In recent years, however, increasing evidence has highlighted the substantial environmental impacts associated with food production, adding a new dimension to the purpose and development of FBDG [6,7]. This shift is reflected in the most recent version of the Nordic Nutrition Recommendations 2023 (NNR2023), which, for the first time, integrated environmental sustainability when determining recommended intake ranges for selected food groups [8]. Healthy and environmentally sustainable FBDG aims to reduce overall environmental impacts, with the dietary carbon footprint (CF) being one of the most used metrics. These global guidelines promote lower consumption of foods from animal sources, particularly red meat, and encourage more nutrient dense, diverse plant-based options [[9], [10], [11]]. These FBDG worldwide are developed for adults. Children’s energy requirements and food intake are lower than those of adults, resulting in lower dietary CF [12]. However, the difference cannot be inferred from energy intake alone, as children’s dietary composition may differ considerably from that of adults [13], and such differences can also vary between countries with otherwise similar dietary cultures [14]. Research on the CF of children’s diets remains limited. Because children’s nutritional needs are different from adults, further research is needed through a child’s lens. Similarly, food systems and cultures vary, so more information is needed about CF in different settings. Filling this gap is important because dietary habits are established in early childhood and understanding them is critical for aligning future population behaviors with current dietary guidelines and global climate goals [12,15].

A recent study by Guðmannsdóttir et al. [16] estimated the dietary CF of adults to be 6.3 kg CO_2_-eq/d, based on data from the Icelandic National Dietary Survey 2019–2021 merged with emission factors from the Danish Big Climate Database [16]. The survey revealed no statistically significant changes in dietary habits over the past decade, although consumption of red meat, fruit and vegetables, and dairy trend downward [16,17]. Findings from another recent study show increasing public support for more sustainable food systems where most adults favored increased education in elementary schools about the health and environmental effects of plant-based diets [18]. These perspectives may help facilitate transition toward more plant-based dietary patterns, as internationally advised in most recently developed FBDG.

The current study aims to perform a secondary analysis of population-based dietary studies among children in Iceland to estimate the CF of children’s diet. The primary objective was to estimate the CF of children’s diets and identify the main food groups contributing to dietary CF across different age groups from 3-y-olds to 15-y-olds. A second objective was to estimate how adherence to the FBDG, adapted to the children’s ages, might alter the CF of major food groups.

## Methods

### Participants and dietary intake

The present study uses dietary intake data previously reported in Thorsdottir et al. [19], derived from most recent population-based surveys of Icelandic children aged 3–15 y. Table 1 provides an overview of these surveys, including sampling strategies and dietary assessment methods.

**TABLE 1:** Dietary surveys of Icelandic children aged 3–15 y included in the study, including study periods, sampling strategies, sample size and dietary assessment methods [[20], [21], [22]]

Age (y)	Study years	Sample description	Complete dietary records (*n*)^1^	Dietary assessment
3	2007	Random sample from the Icelandic Population Registry; capital area	225	3-d weighed food record
5			231	
6	2011–2012	Random national sample	162	3-d weighed food record
9	2003–2004	Nationwide cluster sampling of schools	175	Two 24-h recalls
15			150	

1 Children aged 3 and 5 y *N* = 898 invited, 51% complete records; children aged 6 y *N* = 219 invited, 74% complete records; children aged 9 and 15 y *N* = 183 in each age group, 96% and 82% complete records, respectively.

The study population included 943 participants surveyed between 2003 and 2012, either from the capital area or from across the country [19]. Random sampling from the Icelandic Population Registry was conducted for 3- and 5-y-olds in the capital area (N = 898; 51% complete records) and for 6-y-olds nationwide (N = 219; 74% complete records). Random cluster sampling of schools in all 4 national regions was used to recruit classes of 9- and 15-y-old children (N = 366; 89% complete records). Number of participants were defined as sufficient if >3% of each birth year group of the country’s population participated [23,24] based on prior cohorts it is sufficient to evaluate diet sufficiency [19,[20], [21], [22]].

Dietary intake of the younger children (3-, 5-, and 6-y-olds) was recorded by parent or guardian, at home and in kindergarten, and by research staff in school at after-school daycare, over 3 consecutive days (2 weekdays and 1 weekend day) using a 3-d weighed food record, supplemented with standard portion size estimates when weighing was not feasible [20,21]. Dietary intake of the 9- and 15-y-old participants was assessed using two 24-h recalls, conducted on randomly selected school days (Monday–Friday) through in-person interviews taken by trained research staff about the intake on randomly selected days (Monday–Thursday and Sunday), with the aid of food photographs depicting different portion sizes [22]. Possible misreporting or noncomplete dietary records were defined when mean energy intake from the diet was one third or less of the estimated energy requirement or 3 times or more than the requirement for individuals 9- and 15-year-olds, and for 3- to 6-year-olds age- and sex-specific reference values.

### Estimation of the CF

Dietary intake data were used to estimate the dietary CF using life cycle assessment (LCA) data from the Danish CONCITO Big Climate Database [25], which has been shown to be comparable with corresponding databases in France and the United States when applied to the same dietary intake data when estimating the total dietary CF and food group contribution [16]. The CONCITO database provides CF estimates at the retail level for ∼500 food items found in European grocery stores, including those in Iceland. These estimates quantify the CF in (kg CO_2_-eq) associated with producing 1 kg of each food item and were generated using an input-output analysis and a hybrid consequential LCA. Detailed description regarding the LCA can be found in the methodology report [25]. All recorded dietary data, including food items, quantities, and composite dishes (as recipes), were entered into the ICEFOOD software (Unit for Nutrition Research, University of Iceland), designed for nutrition research and linked to the Icelandic Food Composition Database [[20], [21], [22]]. The CF variable was incorporated into the food composition database as an additional numeric parameter.

**FIGURE 1:**
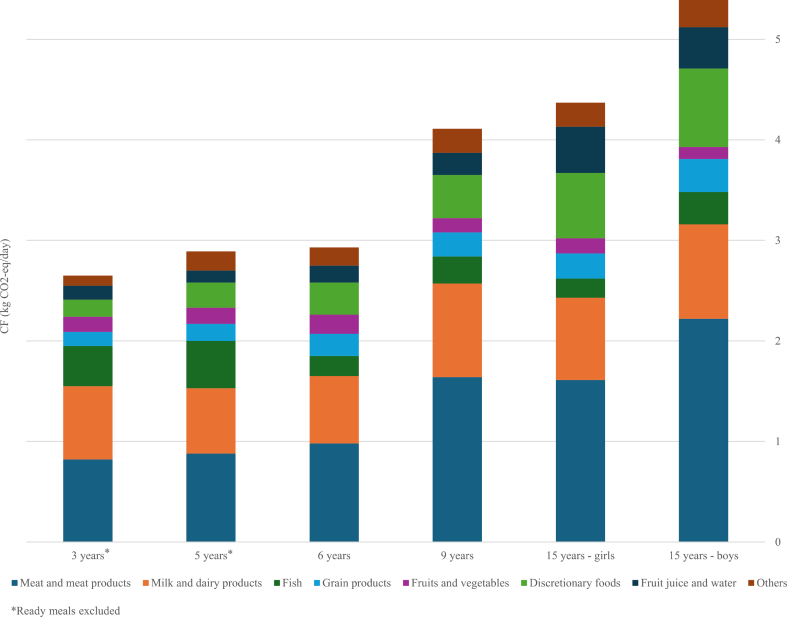
Mean dietary carbon footprint (CF; kg CO_2_-eq/d) from daily food consumption across age groups. Stacked bars show the contribution of food groups to total dietary CF.

**FIGURE 2:**
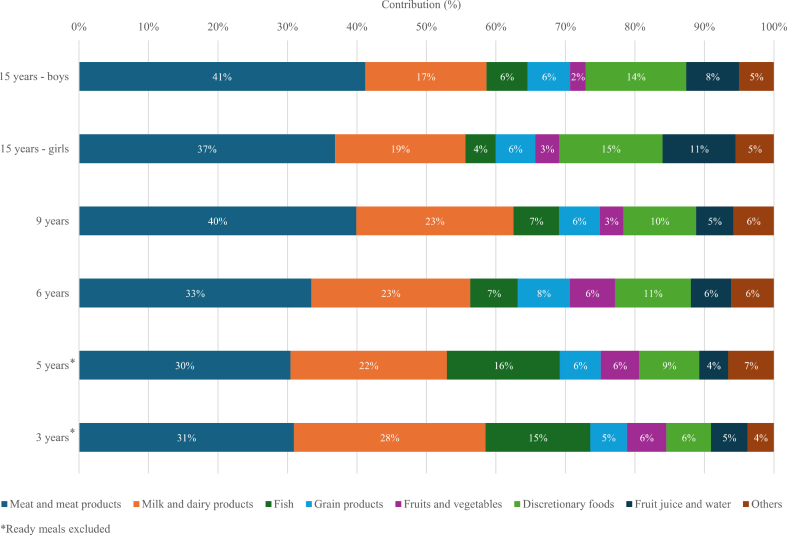
Relative contribution (%) of food groups to total dietary carbon footprint (CF) across age groups.

Mean intake of each food group was calculated for each age group of children. Total CF values were derived by linking the mean consumption of each food group to its respective CF coefficient (kg CO_2_-eq/kg food) from the Danish database [16,25]. In the dietary surveys of 3- and 5-year-olds, a miscellaneous food group labeled “ready meals” of unknown recipes was included but comparable food group was not present in the dietary data for the older age groups of children. To ensure comparability, “ready meals” were excluded from the comparison of total dietary CF estimates as presented in FIGURE 1, FIGURE 2. Total CF values including “ready meals” based on estimated table values are also shown for the youngest age group (Supplementary Material). Changes in food weight during preparation (e.g., water loss or gain through cooking) were accounted for. All CF estimates are adjusted for food loss and waste up to retail level and refer to the amount of edible food consumed. The methodology for the CF estimates has been described elsewhere [16].

Dietary CF per 1000 kcal was estimated for each age group based on the mean energy intakes from the dietary surveys [[20], [21], [22]] combined with the total CF values calculated for this study.

To facilitate comparison with the new FBDG NNR2023 [8,26], CF was also calculated as a hypothetical benchmark for the recommended amounts of 4 key food groups. Recommended amounts for children were derived proportionally from adult recommendations, using the ratio of children’s energy requirements relative to adults [19] as shown in Supplementary Table 1. Recommended daily intake range for children for the 3 food groups, milk and dairy, fish, and fruits and vegetables, are shown in Supplementary Table 1 as well as the CF benchmark based on the range midpoint. For the fourth food group, red meat, according to the guidelines, there is no range for daily or weekly meat intake, only a recommended upper threshold. Therefore, for meat, an upper limit of intake was calculated and used as benchmark for the children’s age groups.

### Ethical approval

All dietary surveys were approved by the Icelandic Data Protection Authority (S2449/2005, S3267/2007, and 2010111049AMK) and by the National Bioethics Committee (VSNb200605002&03, VSNb2005040019/037, and VSNb2011010008/37). Written informed consent was obtained from parents or legal guardians, as well as from the relevant preschool and school committees. Overall findings were presented at parent meetings at schools. This study is reported in accordance with the STROBE-nut reporting guidelines [27].

## Results

Figure 1 shows the mean dietary (CF; kg CO_2_-eq/d) of selected food groups across the 6 groups, demonstrating an increase in dietary CF with age. Mean total dietary CF was 2.9–3.2 kg CO_2_-eq/d among the 3-, 5-, and 6-y-olds and 4.1 kg CO_2_-eq/d for 9-y-olds. Among 15-y-olds, mean total CF was 4.4 kg CO_2_-eq/d for girls and 5.4 kg CO_2_-eq/d for boys, with the highest values observed among boys.

Meat and meat products were the main contributors to dietary CF in all age groups (Figure 1). Among the younger children (3-, 5-, and 6-y-olds), the CF attributed to meat and meat products ranged from 0.8 to 1.0 kg CO_2_-eq/d. In the older groups, total contributions from red meat and meat products were 1.6 kg CO_2_-eq/d for 9-y-olds and 15-y-old girls, and 2.2 kg CO_2_-eq/d for 15-y-old boys, with red meat alone accounting for 1.2 kg CO_2_-eq/d in 9- and 15-y-old girls, and 1.6 kg CO_2_-eq/d for 15-y-old boys. Milk and dairy products were the second-largest contributor, accounting for 0.7–0.9 kg CO_2_-eq/d across all age groups, when including milk, cocoa milk, cheese and other dairy products, but was diminished to 0.6–0.8 kg CO_2_-eq/d when excluding cheese (Supplementary Table 2A and B).

The third contributor to total dietary CF varied between age groups, being fish or discretionary foods, whereas contributions from other food groups were substantially lower. Detailed CF values for total diets and for selected food groups across age groups are provided in Supplementary Table 2A and B.

Dietary CF per 1000 kcal ranged from 1.9–2.1 kg CO_2_-eq/1000 kcal among the youngest age groups (3-, 5-, and 6-y-olds), 2.1 kg CO_2_-eq/1000 kcal among 9-y-olds and 2.0 kg CO_2_-eq/1000 kcal and among 15-y-old boys and girls.

Figure 2 describes the proportional contribution of major food groups to total dietary CF across age groups. Meat and meat products together with milk and dairy products as primary contributors among all age groups, together ranging from 52% to 63% of total dietary CF. Fish was the third-largest contributor among 3- and 5-y-olds, contributing 15% and 16% of the total CF. Fish contributed less to dietary CF with increasing age, declining to 4% among adolescent girls. In contrast, discretionary foods, including ice cream, chips and popcorn, candy, sugar and honey, biscuits and cakes, and soft drinks, emerged as the third-largest contributor to total dietary CF among children aged ≥6 y. Discretionary foods contributed 15% of total CF among 15-y-old girls and 14% among boys and accounted for 10% and 11% among 9- and 6-y-olds, respectively. Among the youngest children, discretionary foods contributed 6% of total CF in 3-y-olds and 9% in 5-y-olds. The proportion of total CF from grains ranged between 5% and 8% across all age groups. Contribution from fruit juice varied and was highest among 15-y-old adolescents, 11% and 8% for girls and boys, respectively. Vegetables and fruits contributed only a few percent to the total CF (2%–3%), and more among the younger age groups (3-, 5-, and 6-year-olds) or 6%. Other foods, including eggs, pizza, fries and potatoes, gravy, and animal fat, contributed 4%–7% to the total CF across all age groups.

**FIGURE 3:**
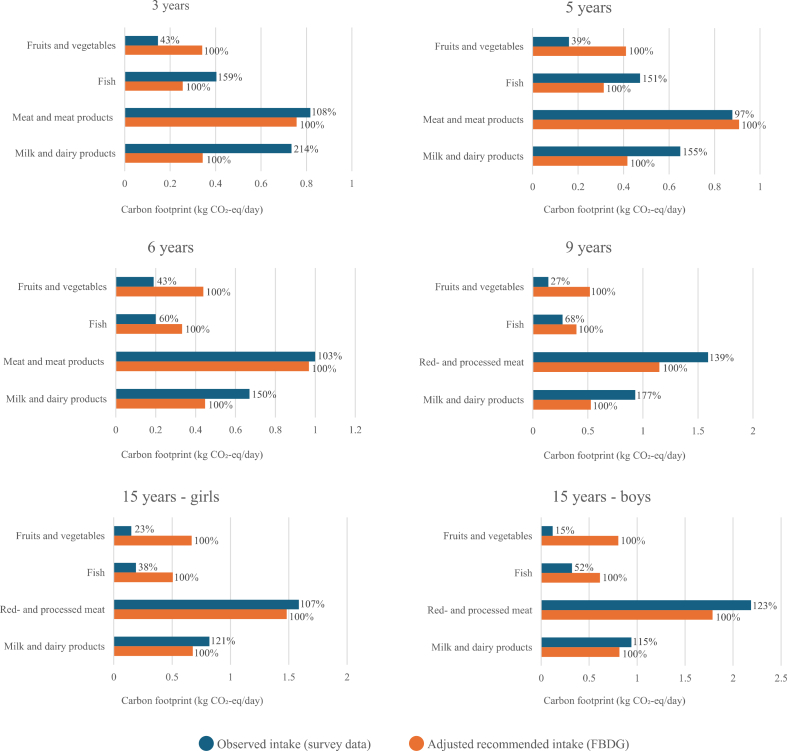
Comparison of observed dietary carbon footprint (CF) with CF calculated from energy-adjusted food-based dietary guidelines (FBDG) for each age group. Percentages indicate the deviation of observed CF from recommended intakes.

Figure 3 compares the estimated CF associated with the recommended intake of 4 main food groups, based on FBDG for adults, calibrated for children’s energy requirements with the observed CF from the children’s actual diets. The percentage values indicate how the observed dietary CF differed from the CF estimated for the recommendations. Across all age groups, the CF from fruits and vegetables was consistently much lower than the calculated CF from the recommended midpoint intake level, ranging from 15% to 43%, with the highest proportion observed among the youngest children. In contrast, CF from meat and meat products was generally at or above the total dietary CF corresponding to the maximum recommended intake of red meat.

Milk and dairy products consistently exceeded the calculated CF levels for recommended intake range, based on the midpoint of the recommended intake; this was particularly evident in younger age groups, where it ranged from more than double among 3-y-olds to 50% higher among 6-y-olds. The CF from fish intake was relatively high among the youngest children (3- and 5-y-olds) as compared with that from recommended intake, but was notably lower among adolescents, especially girls.

## Discussion

This study examined the dietary CF of Icelandic children across 5 age groups, from 3 to 15 y, using data collected during the first 2 decades of the 21st century. Participants were randomly selected either from the Reykjavík capital area or nationwide [19]. Estimating the CF of children’s diets across age groups provides a crucial baseline for evaluating future dietary surveys, which are planned in Iceland for 2026–2029.

The total CF of the diet was highest among 15-y-old boys (5.4 kg CO_2_-eq/d) and was ∼25% higher than among girls of the same age (4.4 kg CO_2_-eq/d). The CF among 9-y-olds (4.1 kg CO_2_-eq/d) was substantially higher than in the 3 younger age groups (≈3.0 kg CO_2_-eq/d). Notably, the CF of 9-y-olds was similar to that of 15-y-old girls, whereas the 3 youngest age groups exhibited comparable total CF values.

### Comparison with other Nordic and European studies

Recently published studies from other Nordic countries have reported lower CF values for children’s diets than observed in this study [12,28,29]. For example, Wright et al. [12] estimated a CF of 2.1 kg CO_2_-eq/d for Norwegian 2-y-olds, and Jacobsen et al. [29] reported 2.3 kg CO_2_-eq/d for 4-y-olds in Sweden. Furthermore, the estimated CF for Icelandic adolescents were ∼1.0 kg CO_2_-eq/d (25%–30%) higher than for Swedish adolescents [28]. These discrepancies likely reflect differences in dietary habits, such as higher meat and dairy intake in Iceland, as well as the earlier time-period of Icelandic data collection (∼10–13 y before the Nordic collections). Variations in the LCA data sources used for CF calculations may contribute to these discrepancies to varying degrees. Previous studies [30,31], demonstrate that different LCA methodologies can result in ∼10% discrepancy in total CF estimates. However, Guðmannsdóttir et al. [16] observed an even smaller variation once system boundaries were harmonized across the LCA databases using the same food intake data.

In the German Dortmund Nutritional and Anthropometric Longitudinally Designed (DONALD) study, which included children aged 6–17 y (mean age 11–12 y) between 2000 and 2021, the mean dietary CF was 4.0 kg CO_2_-eq/d, corresponding to 2.3 kg CO_2_-eq/1000 kcal [32]. The primary contributors were meat products (25.6%), dairy products (22.2%), and sweets and pastries (14.0%). In comparison, dietary CF in our findings ranged from 1.9 to 2.1 kg CO_2_-eq per 1000 kcal across children aged 3–15 y, which is slightly lower but comparable with the DONALD study.

### Main contributors to dietary CF

In the present study, the contribution of different food groups to total CF varied somewhat by age. Across all age groups, meat and dairy products remained the dominant contributors. Meat and meat products accounted for ∼30%–41% of total dietary CF among all the age groups of children, with red and processed meat being the main contributors. Although poultry has a lower CF per kilogram than red meat [10,25,30], its minimal contribution in this study also reflects the notably low consumption at the time of data collection. Dairy products accounted for 17%–19% of the dietary CF among adolescents, and 22%–28% among younger children. Discretionary foods accounted for a substantial proportion of CF among adolescents and older children, accounting for 10%–15% of the dietary CF of 6- to 15-y-olds boys and girls, but were less prominent in youngest children’s diets, though still 6% and 9% of the dietary CF of 3- and 5-y-olds.

When comparing the results with the maximum recommended intake of red meat, the CF estimates for most age groups were below this limit. However, when including processed meat, the intake clearly exceeded the CF of the recommended maximum red meat intake. According to the Icelandic National Dietary Survey 2019–2021 on adults, the CF from red meat was considerably higher than the values observed in the present study of children. The adult CF estimates exceeded the CF of maximum recommended level by 75% [8,16,17,26]. Considering dairy products, the CF among adults was only 45% of the value corresponding to the midpoint recommended intake, which is lower than that observed among children. Fish showed an 11% higher CF than corresponding value for midpoint recommended intake for adults, this is a value that varies among the age groups of children, with dietary CF lower than calculated from the recommendation for most age groups but quite high for the 2 youngest groups of children. The high CF from fish and high intake of the youngest seem to be based on the intake in kindergartens. Vegetables and fruits were 35% of the total CF among adults, which mirrors the low intake of these foods as found in the age groups of children.

### Implications for dietary change

A recent national survey on Icelandic adults’ perceptions toward plant-based diets revealed broad public support for increasing education about plant-based eating in schools and for providing greater subsidies to farmers producing local plant-based foods [18]. These findings are encouraging considering the relatively high total CF observed in Icelandic children’s diets measured in the past 2 decades, and supports initiatives aimed at promoting more sustainable dietary patterns starting in early childhood.

Previous reports have described the nutrient intake and health characteristics of these study participants [19]. These showed that children had too low or marginally too low intake of several vitamins and minerals [[20], [21], [22]]. Also, the diet was low in dietary fiber and did not have a recommended combination of energy yielding nutrients [8,[20], [21], [22],^26^]. A large part of the energy intake was based on nutrient poor discretionary foods as shown for CF in the current study, being 6%–15% and earlier estimated as percentage of energy being between 11% among 3-y-olds and 25% for adolescents [19]. A recent Finnish study similarly shows a significant proportion of food expenditure is based on discretionary foods and negatively affect both environmental footprints and the nutritional quality of food purchases in households [33]. Consequently, there is need to improve the nutritional value of the diet. It has been debated whether children should follow the same relative intake of milk and dairy products as adults or if they need a higher intake [19,34].

A recent Danish study demonstrated a 15% reduction in CF following environmentally focused procurement changes in preschools, primarily through a 37% decrease in ruminant meat purchases, whereas maintaining nutritional adequacy [34]. Further reductions in dairy were not recommended, as requirements for children for some nutrients, e.g., calcium remained difficult to meet even after dietary adjustments. Importantly, preschools that received targeted dietary guidance achieved 14% lower CF by 2022 in comparison with 2018, aligning with Copenhagen’s municipal goal of a 25% reduction by 2025.

A Dutch study similarly found that adherence to national FBDG was associated with substantially lower CF among both adults and children, largely due to reduced consumption of red and processed meat and increased intake of vegetables [35]. The present findings are consistent with these results and with the Icelandic National Dietary Survey 2019–2021 [17], suggesting that dietary shifts toward reduced meat and increased plant-based food consumption of high quality can simultaneously improve health and environmental outcomes.

In Sweden, optimized school lunches have demonstrated that CF can be reduced substantially without compromising nutritional adequacy [[36], [37], [38]]. The baseline CF of an average Swedish adolescent’s diet (4.5 kg CO_2_-eq/d) was comparable to that of 15-y-old girls in our study, indicating realistic potential for improvement in Iceland through similar school-based strategies.

Numerous studies have confirmed among adults that meat, dairy (particularly cheese), discretionary foods, and beverages are major contributors to dietary CF, whereas CF is typically lower among children than adults and among females compared with males, patterns also evident in our data [29,30,35,39,40]. The CF of Icelandic children’s diets was slightly higher than in comparable countries. The dietary CF patterns resembled those of Icelandic adults in 2019–2021 (per energy unit), though with lower meat intake, higher intake of dairy and discretionary foods for some of the age groups, whereas all, both adults and children have low intake of vegetables and fruits, as well as grains. A transition to high quality, more plant-based diet which is in line with recent FBDG is feasible to improve the CF and nutrient intake of children and adults [8]. It is also noteworthy that according to results from the Malmö Diet and Cancer Cohort a more climate-friendly diet had a lower intake of micronutrients but did not substantially increase risk of deficiencies [41].

### Strengths and limitations

The main strengths of this study include the use of randomly selected participants, detailed dietary data collected with traditional and validated methods, and realistic energy intake of participants [19] and CF estimation based on internationally comparable LCA data [25]. A country-specific database should most often be preferred and the CF of foods in the Danish database might differ from foods on the Icelandic market, where green energy is used in food production. However, the usage of CONCITO is a valid approach considering Iceland’s high volume of food imports and the comparability of its domestic agricultural emissions to the Nordic region [42]. It could also be argued in this case that using internationally comparable LCA data is a strength rather than a limitation, as it reflects the reality of Iceland’s dependence on food and feed imports. A primary limitation is the data being >10–20 y; however, no other data are available, and these results provide an invaluable baseline for assessing forthcoming national dietary surveys of Icelandic children and for evaluating temporal trends in dietary CF. Furthermore, a recent survey on diet among adults shows no statistically significant changes in dietary habits over the past decade, although consumption of red meat, fruit and vegetables, and dairy trend downward [16,17]. Although more plant-based foods are available on the market, a study reported a low prevalence of vegans [43]. Similar results have been observed in other Nordic countries [44]. Another limitation is the use of varied dietary assessment methods across age groups representing different days of the week, i.e., 4 possible weekdays and 1 weekend day among 9- and 15-y-olds compared with always 2 weekdays and 1 weekend day among younger children. Because dietary intake may vary between weekdays and weekends, and the methodology differs by age group, day measured could potentially contribute to differences found between age groups. This means probable higher intake of discretionary foods of the 9- and 15-y-olds than shown in the results and therefore also higher CF from the discretionary foods and a larger difference from the younger age groups. The original data collection among the 5 age groups of children defined food types slightly differently in the various studies which were the basis for this current secondary analysis, e.g., including or not “ready meals” as a specific food type [[20], [21], [22]]. This problem was solved by using experience between the studies or from other cohorts [17]. Finally, CF is only one estimate of an environmentally sustainable diet, and other metrics, such as all costs related to food importation, should also be considered in future studies.

In conclusion, this study highlights substantial contributions of meat and discretionary foods to children’s dietary CF across all age groups, indicating clear opportunities for shifting toward a more environmentally sustainable diet. The present findings underscore the need for continued education, supportive policy measures, and integration of sustainability goals into dietary planning for children and adolescents. Encouragingly, recent public opinion [18] in Iceland supports such changes, providing momentum for sustainable dietary transformation in the years to come. A dietary transition toward the FBDG, including a more plant-based eating in Iceland would benefit both children’s health and the environment by aligning food consumption more closely with health-based recommendations and reducing dietary CF.

## Author contributions

The authors’ responsibilities were as follows – TO, RG, IT: designed the research, prepared the data and drafted the main manuscript text; RN, BT: contributed to preparation of data analysis and critically reviewed the draft of the manuscript; TO, RG: performed the data analysis; IT: had primary responsibility for final content; and all authors: read and approved the final manuscript.

## Data availability

Data described in the manuscript are not publicly available but can be made available upon reasonable request, pending application and approval from the institutions responsible for the original dietary surveys.

## Declaration of generative AI and AI-assisted technologies in the writing process

The author(s) declare that no generative AI or AI-assisted technologies were used in the writing of this manuscript.

## Funding

This study was supported by the Research Fund of the University of Iceland (to IT and BT). Former supporters were the Directorate of Health and Public Health Institute of Iceland, Science Fund of Landspitali University Hospital and the Icelandic Research Fund (to IT).

## Conflict of interest

The authors declare that they have no conflicts of interest.
