# Supplementary material for: Carbon Footprint of Childhood Diets—A Secondary Analysis of Population-Based Studies
Source: Curr Dev Nutr. 2026 Apr 21;10(6):107705. doi: 10.1016/j.cdnut.2026.107705 (PMC13207462; doi:10.1016/j.cdnut.2026.107705)
Supplement: multimedia component 1 [file mmc1.pdf]

Supplementary Table 1. Food based dietary guidelines (FBDG) as estimated amounts for children in the respective age groups derived from FBDG for adults in the Nordic Nutrition Recommendations (NNR) according to the ratio of energy requirements for each age group relative to adult requirements. Intake ranges (g/day) and corresponding midpoints (g/day) are shown for fruits and vegetables, fish and milk and dairy products. For red meat, the recommended intake, maximum, is shown (g/day). Carbon footprint (CF) benchmarks are estimated as calculated for midpoints and maximum intake.

						Girls	Boys
**Food groups**	**Adults**	**3 years**	**5 years**	**6 years**	**9 years**	**15 years**	**15 years**
Fruits and vegetables							
Range (g/day)	500–800	250–400	302–483	323–516	380–607	490–784	590–944
Midpoint (g/day)	650	325	393	420	494	637	767
CF (kg CO_2-eq/day)	0.68	0.34	0.41	0.44	0.52	0.67	0.80
Fish							
Range (g/day)	43–64	21–32	26–39	28–41	33–49	42–63	51–76
Midpoint (g/day)	54	27	33	35	41	53	64
CF (kg CO_2-eq/day)	0.52	0.26	0.31	0.33	0.40	0.51	0.61
Milk and dairy products							
Range (g/day)	350–500	174–248	211–302	226–323	266–380	342–490	413–590
Midpoint (g/day)	425	211	257	275	323	416	502
CF (kg CO_2-eq/day)	0.69	0.34	0.42	0.45	0.53	0.68	0.82
Red meat							
Maximum (g/day)	50	25	30	32	38	49	59
CF (kg CO_2-eq/day)	1.52	0.76	0.91	0.97	1.15	1.48	1.79
