# Supplementary material for: Carbon Footprint of Childhood Diets—A Secondary Analysis of Population-Based Studies
Source: Curr Dev Nutr. 2026 Apr 21;10(6):107705. doi: 10.1016/j.cdnut.2026.107705 (PMC13207462; doi:10.1016/j.cdnut.2026.107705)
Supplement: multimedia component 2 [file mmc2.pdf]

Supplementary Table 2a. Mean daily consumption (g/day) and dietary carbon footprint (CF; kg CO_2-eq/day) of food groups among children aged 3, 5 and 6 years.

**Food groups**	**3 years**		**5 years**		**6 years**	
	Consumption Mean (SD) g/day	CF, Mean (SD) kg CO_2-eq/day	Consumption Mean (SD) g/day	CF, Mean (SD) kg CO_2-eq/day	Consumption Mean (SD) g/day	CF, Mean (SD) kg CO_2-eq/day
Milk and dairy products (total)					357 (193)	0.58 (0.31)
Milk	342 (190)	0.48 (0.26)	306 (172)	0.39 (0.22)		
Cocoa milk	42 (67)	0.04 (0.07)	49 (72)	0.05 (0.07)		
Dairy products other	112 (90)	0.16 (0.13)	106 (93)	0.15 (0.13)		
Cheese	10 (9)	0.05 (0.05)	11 (10)	0.06 (0.05)	17 (14)	0.09 (0.07)
Eggs	3 (10)	0.003 (0.01)	3 (8)	0.003 (0.01)		
Ice cream and candy (total)					32 (33)	0.09 (0.09)
Candy, sugar and honey	5 (9)	0.01 (0.02)	9 (13)	0.02 (0.03)		
Ice cream	12 (22)	0.03 (0.05)	14 (24)	0.03 (0.05)		
Chips and popcorn	2 (4)	0.01 (0.01)	3 (8)	0.01 (0.02)	8 (14)	0.02 (0.04)
Bread	60 (30)	0.08 (0.04)	78 (36)	0.10 (0.05)	80 (39)	0.10 (0.05)
Biscuits	7 (8)	0.02 (0.03)	8 (11)	0.03 (0.04)	13 (18)	0.04 (0.06)
Cakes	20 (27)	0.06 (0.09)	35 (37)	0.11 (0.12)	32 (34)	0.10 (0.11)
Cereals	19 (15)	0.03 (0.03)	23 (19)	0.04 (0.03)	43 (38)	0.08 (0.07)
Oatmeal					16 (36)	0.02 (0.04)
Pasta	21 (32)	0.03 (0.04)	23 (39)	0.03 (0.05)	19 (29)	0.02 (0.04)
Pizza	12 (26)	0.05 (0.11)	14 (25)	0.06 (0.10)	12 (28)	0.05 (0.12)
Vegetables	32 (32)	0.04 (0.04)	44 (40)	0.05 (0.04)	52 (47)	0.06 (0.05)
Potatoes	35 (29)	0.06 (0.05)	38 (28)	0.06 (0.05)	21 (28)	0.04 (0.05)
Fries					6 (12)	0.03 (0.05)
Fruits and berries	112 (70)	0.11 (0.07)	112 (76)	0.11 (0.08)	136 (102)	0.13 (0.10)
Meat and meat products, total	54 (37)	0.82 (0.56)	58 (42)	0.88 (0.64)	65 (35)	0.98 (0.53)
Fish and fish products	42 (33)	0.40 (0.32)	49 (34)	0.47 (0.33)	21 (24)	0.20 (0.23)
Butter and margarine	6 (5)	0.02 (0.02)	8 (6)	0.03 (0.02)	9 (8)	0.03 (0.03)
Dressings	9 (11)	0.02 (0.03)	11 (15)	0.03 (0.04)	13 (11)	0.03 (0.03)
Cod liver oil	2 (2)	0.01 (0.01)	2 (2)	0.01 (0.01)	2 (3)	0.01 (0.01)
Soft drinks	48 (87)	0.03 (0.05)	56 (82)	0.04 (0.05)	71 (99)	0.04 (0.06)
Soda	13 (34)	0.01 (0.01)	32 (56)	0.01 (0.02)	46 (76)	0.02 (0.03)
Fruit juice	67 (85)	0.11 (0.14)	58 (85)	0.09 (0.14)	87 (107)	0.14 (0.17)
Water and mineral water	267 (190)	0.03 (0.02)	289 (181)	0.03 (0.02)	285 (235)	0.03 (0.03)
Ready meals	64 (58)	0.27 (0.24)	71 (71)	0.30 (0.30)		
**Total dietary carbon CF**		**2.97 (0.82)**		**3.18 (0.88)**		**2.94 (0.74)**

CO_2-eq = Carbon dioxide equivalent

Supplementary Table 2b: Mean daily consumption (g/day) and dietary carbon footprint (CF; kg CO_2-eq/day) of food groups among children aged 9 years and 15-year-old girls and boys.

**Food groups**	**9 years**		**15 years - girls**		**15 years - boys**	
	Consumption Mean (SD) g/day	CF, Mean (SD) kg CO_2-eq/day	Consumption Mean (SD) g/day	CF, Mean (SD) kg CO_2-eq/day	Consumption Mean (SD) g/day	CF, Mean (SD) kg CO_2-eq/day
Milk and dairy products	570 (292)	0.81 (0.42)	479 (297)	0.66 (0.41)	545 (350)	0.73 (0.47)
Cheese	22 (19)	0.12 (0.10)	30 (20)	0.16 (0.11)	40 (31)	0.21 (0.16)
Eggs	4 (10)	0.003 (0.01)	6 (18)	0.01 (0.02)	9 (16)	0.01 (0.01)
Ice cream	12 (30)	0.03 (0.07)	21 (44)	0.05 (0.10)	11 (29)	0.03 (0.07)
Chips and popcorn	6 (13)	0.02 (0.03)	8 (15)	0.02 (0.04)	16 (29)	0.04 (0.08)
Candy, sugar and honey	17 (20)	0.04 (0.05)	34 (29)	0.08 (0.07)	39 (50)	0.09 (0.11)
Bread	123 (63)	0.15 (0.08)	140 (75)	0.18 (0.09)	179 (103)	0.22 (0.13)
Biscuits and cakes	48 (52)	0.16 (0.17)	80 (72)	0.26 (0.23)	83 (79)	0.27 (0.26)
Cereals	36 (39)	0.06 (0.07)	27 (45)	0.05 (0.08)	38 (46)	0.07 (0.08)
Pasta	19 (34)	0.02 (0.04)	21 (48)	0.03 (0.06)	32 (57)	0.04 (0.07)
Pizza	24 (46)	0.10 (0.19)	28 (55)	0.12 (0.23)	29 (72)	0.12 (0.30)
Vegetables	45 (50)	0.05 (0.06)	56 (48)	0.06 (0.05)	51 (46)	0.06 (0.05)
Potatoes	44 (51)	0.07 (0.09)	40 (55)	0.07 (0.09)	53 (60)	0.09 (0.10)
Fries	5 (13)	0.02 (0.05)	5 (12)	0.02 (0.05)	5 (14)	0.02 (0.06)
Fruits and berries	94 (92)	0.09 (0.09)	86 (114)	0.09 (0.11)	57 (64)	0.06 (0.06)
Meat and meat products:						
Red meat	38 (45)	1.15 (1.36)	38 (44)	1.15 (1.33)	53 (55)	1.60 (1.66)
Poultry	17 (32)	0.04 (0.08)	8 (20)	0.02 (0.05)	11 (29)	0.03 (0.08)
Processed meat	31 (50)	0.44 (0.80)	32 (54)	0.44 (0.86)	43 (58)	0.59 (0.91)
Fish and fish products	28 (40)	0.27 (0.39)	20 (32)	0.19 (0.31)	33 (46)	0.32 (0.44)
Fats (butter, oils, dressings)	14 (11)	0.04 (0.03)	11 (10)	0.03 (0.03)	11 (10)	0.03 (0.03)
Coffee, tea and cocoa powder	18 (42)	0.01 (0.01)	22 (62)	0.01 (0.02)	22 (53)	0.01 (0.02)
Soft drinks and soda	349 (301)	0.19 (0.16)	452 (470)	0.25 (0.26)	641 (495)	0.35 (0.27)
Fruit juice	85 (141)	0.16 (0.27)	200 (257)	0.38 (0.49)	174 (217)	0.33 (0.42)
Water and mineral water	418 (275)	0.05 (0.03)	616 (381)	0.07 (0.04)	625 (444)	0.07 (0.05)
**Total dietary carbon CF**		**4.11 (1.65)**		**4.37 (1.70)**		**5.39 (2.03)**

CO_2-eq = Carbon dioxide equivalent
